# A Heart Under Stress: Anaesthetic Strategy for a Pregnant Patient With Long QT Syndrome

**DOI:** 10.7759/cureus.100633

**Published:** 2026-01-02

**Authors:** Maria J Nascimento, Marta Silva, Ana Bernardino, Joana Carvalhas

**Affiliations:** 1 Anesthesiology, Unidade Local de Saúde de Coimbra, Coimbra, PRT

**Keywords:** anaesthetic challenges and management, congenital long qt syndrome, general anaesthesia, obstetrics anaesthesia, peripartum management, ventricular dysrhythmia

## Abstract

Long QT syndrome (LQTS) is a cardiac channelopathy associated with a high risk of malignant arrhythmias. Hormonal changes during pregnancy, especially in the peripartum period, increase the risk of adverse cardiac events in women with LQTS. Anaesthetic management in these patients is challenging, as there are no established guidelines and most anaesthetic agents prolong the QT interval.

This report describes the anaesthetic management of an elective caesarean section in a 34-year-old pregnant woman, 35 weeks pregnant, with congenital LQTS type 1 (LQTS1), a corrected QT interval (QTc) of 597 milliseconds (ms) on the admission day, and a high cardiovascular risk. Given the high arrhythmic risk, perioperative planning and management were undertaken by a multidisciplinary team to optimise patient outcomes and minimise risks. Total intravenous anaesthesia with propofol and remifentanil was used, alongside strict electrolyte optimisation, sympathetic control, and continuous advanced monitoring.

Careful perioperative planning and appropriate monitoring can enable the safe use of general anaesthesia for caesarean section in selected women with LQTS1.

## Introduction

Long QT syndrome (LQTS) is a life-threatening cardiac channelopathy caused by abnormal ion channel function, leading to prolongation of ventricular repolarisation and predisposing affected individuals to malignant arrhythmias such as polymorphic ventricular tachycardia (VT) [1]. These arrhythmias may result in syncope or sudden cardiac death (SCD) [1]. LQTS affects approximately 1 in 2,000 individuals and is primarily inherited [1]. Approximately 80-90% of cases are linked to mutations in three specific genes - KCNQ1 (LQTS1), KCNH2 (LQTS2), which are potassium-channel genes, and SCN5A (LQTS3), a sodium-channel gene [2,3]. Congenital LQTS may manifest at any stage of life and can be detected from the foetal period to adulthood [4].

Diagnosis relies on electrocardiographic findings, clinical presentation and genetic analysis [3]. A prolonged QT interval on an electrocardiogram (ECG) is the key indicator for an LQTS diagnosis. The normal range of QTc differs by age and sex, with prolonged QTc being defined as >470 ms in women older than 15 years [5]. Women with LQTS generally exhibit longer QT intervals and a higher risk of VT or SCD compared with men, largely due to hormonal fluctuations throughout the menstrual cycle, pregnancy and the postpartum period. Consequently, pregnancy and the peripartum period are associated with an increased arrhythmic risk in this population [1,2,5].

While cardiovascular management of pregnant patients with LQTS is relatively well-described, specific guidelines for anaesthetic management remain unavailable [1,3]. Published literature on anaesthetic management in pregnant women with congenital LQTS is limited to small case series and isolated case reports, with no consensus regarding the optimal anaesthetic technique, particularly in high-risk patients [3]. Given the lack of established anaesthetic guidelines for these patients, multidisciplinary planning is essential to ensure maternal and foetal safety [1,2].

## Case presentation

A primigravida in her 30s, with a known diagnosis of LQTS1, was hospitalised at 31 weeks of gestation following the identification of an atrioventricular (AV) block on routine foetal echocardiography, raising suspicion of foetal LQTS. She had been under close surveillance throughout pregnancy, including beta-blocker adjustments and electrolyte monitoring.

Her medical history included two cardiac ablations and implantable cardioverter-defibrillator (ICD) implantation due to sustained VT and recurrent cardiogenic syncope, despite beta-blockers and left sympathectomy.

**Figure 1:**
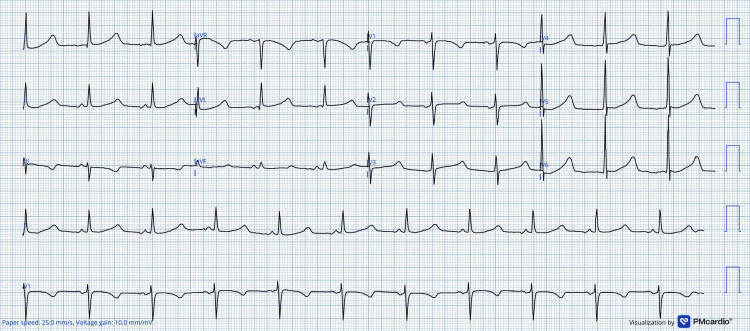
Admission ECG ECG performed on hospital admission showing marked QTc prolongation (QTc 597 ms). A digital ECG converter was used to increase the resolution of the original image.

At admission, her QTc interval was markedly prolonged at 597 ms (Figure 1), necessitating enhanced monitoring and therapeutic adjustments, including an increased beta-blocker dose, the addition of oral spironolactone, and intravenous potassium and magnesium supplementation, due to persistently low serum levels.

A multidisciplinary meeting involving anaesthesiologists, obstetricians and cardiologists concluded that she was a high-risk patient and an elective caesarean delivery at 35 weeks was the safest course of action. This decision aimed to pre-empt the risks of prolonged maternal intravenous ion supplementation, the potential for spontaneous labour triggering life-threatening arrhythmias and recurrent episodes of foetal bradycardia due to AV block.

ICD interrogation confirmed appropriate function. Her last shock occurred at seven weeks of gestation, triggered by anxiety. Multiple anxiety episodes during hospitalisation were managed with alprazolam 1 mg.

**Figure 2:**
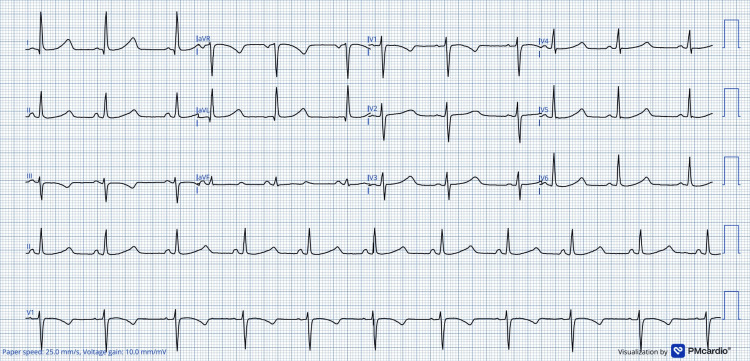
Preoperative ECG ECG obtained following pharmacologic and electrolyte optimisation, demonstrating substantial QTc improvement (QTc 457 ms) prior to elective caesarean delivery. A digital ECG converter was used to increase the resolution of the original image.

Preoperative blood analysis was normal, and therapeutic adjustments reduced her QTc to 457 ms (Figure 2).

On the day of the surgery, American Society of Anesthesiologists (ASA) standard monitoring was implemented. The ICD remained active, with defibrillation pads and a five-lead ECG applied as precaution. An arterial line was placed. Bispectral index (BIS), neuromuscular monitoring (TOF) and urine output were continuously monitored.

Prior to anaesthetic induction, the patient received 2 mg of midazolam and 2 g of magnesium sulphate. A remifentanil infusion was initiated at 0.03 µg/kg/min. Rapid sequence induction was performed using 1 mg/kg lidocaine, 2.5 mg/kg propofol and 1 mg/kg rocuronium. Intubation was successful using videolaryngoscopy.

Anaesthesia was maintained with total intravenous anaesthesia (TIVA) of propofol (target controlled infusion effect-site of 3 µg/mL) and remifentanil titrated to 0.08 µg/kg/min. Dexamethasone 4 mg was administered.

Remifentanil was discontinued three minutes before delivery. The newborn had APGAR (Appearance, Pulse, Grimace, Activity, Respiration) scores of eight, nine, and nine. Post-delivery analgesia included 200 µg fentanyl, 1 g paracetamol, and 30 mg ketorolac. Sulprostone and misoprostol were used as uterotonics.

The patient remained stable, with no arrhythmias. Mild hypokalaemia was corrected intraoperatively. Before extubation, 2 g magnesium sulphate and a bilateral transversus abdominis plane (TAP) block with 0.2% ropivacaine (30 mL per side) were administered. Sugammadex was used for reversal. Extubation occurred under remifentanil 0.02 µg/kg/min.

She was monitored for 6 hours in the post-anaesthesia care unit and then transferred to the cardiac intensive care unit, where she remained for 72 hours. The immediate postpartum period was uneventful. Postoperative pain management included paracetamol and ketorolac.

**Figure 3:**
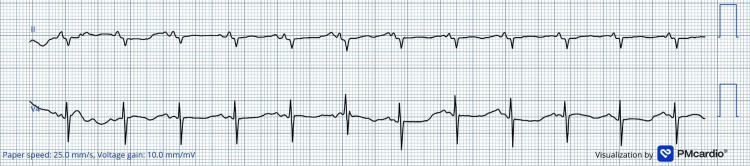
Partial ECG of the newborn Postnatal partial ECG from the newborn, due to poor technical quality, revealing marked QTc prolongation (QTc 600 ms), supporting the diagnosis of congenital LQTS and guiding early therapeutic intervention. A digital ECG converter was used to increase the resolution of the original image.

The newborn's ECG, performed three hours after birth, showed prolonged QTc (Figure 3), and beta-blocker therapy was started. Both mother and baby continued follow-up with cardiology after hospital discharge.

Table 1 summarises the timeline of key clinical parameters, perioperative interventions and outcomes.

**Table 1:** Timeline of key clinical parameters and interventions in this case LQTS1, long QT syndrome type 1; QTc, corrected QT interval; ECG, electrocardiogram; AV, atrioventricular; ICD, implantable cardioverter-defibrillator; IV, intravenous; MgSO₄, magnesium sulfate; TIVA, total intravenous anaesthesia; TAP, transversus abdominis plane; APGAR, Appearance, Pulse, Grimace, Activity, Respiration

Timepoint	QTc / ECG findings	Electrolytes	Key interventions	Clinical outcome
Admission (31 weeks’gestation)	QTc 597 ms; Maternal high-risk LQTS1; Suspected foetal AV block	Persistent low potassium (<3.8 mmol/L) and magnesium (<1.9 mg/dL)	Increased beta-blocker dose; IV potassium and magnesium supplementation; Spironolactone added; Multidisciplinary planning	QTc improved after optimisation
Preoperative period (35 weeks’ gestation)	QTc 457 ms	Optimised or within target range	ICD interrogation; Avoidance of QT-prolonging drugs; Plan for elective caesarean section	Patient prepared for elective caesarean section
Induction and maintenance	Five-lead ECG and defibrillation pads applied as precaution; Arterial line; No arrhythmias reported	Monitored; Mild hypokalaemia corrected intraoperatively	Midazolam 2 mg; MgSO₄ 2 g; Remifentanil infusion; Induction with lidocaine, propofol and rocuronium; Maintenance with TIVA	Stable induction; Successful intubation; Haemodynamically stable
Delivery	No arrhythmias reported	—	Remifentanil stopped 3 minutes before delivery	Neonate APGAR 8/9/9
Post-delivery	No arrhythmias reported	Continued monitoring	Multimodal analgesia with fentanyl, paracetamol, ketorolac and TAP block; Uterotonics: sulprostone and misoprostol; MgSO₄ 2 g	Uneventful emergence and extubation
Postoperative period	Maternal course uneventful	Maintained	Cardiac intensive care unit monitoring for 72 hours	No arrhythmic events
Neonatal assessment	Neonatal ECG with prolonged QTc	—	Beta-blocker therapy initiated	Follow-up with cardiology

## Discussion

Pregnancy-related hormonal changes affect cardiac repolarisation, increasing arrhythmic risk [1,2]. Labour and delivery further elevate this risk through sympathetic stimulation and exposure to QT-prolonging drugs [3,4,6]. 

LQT1 is particularly susceptible to arrhythmias triggered by adrenergic stimulation or emotional stress [1,5]. Beta-blockers are the cornerstone of treatment during and after pregnancy, significantly reducing adverse cardiac events, particularly in LQT1 [3,5]. In high-risk cases, ICD placement and left cardiac sympathetic denervation are adjunctive strategies [3].

Preoperative evaluation and multidisciplinary planning are critical for anaesthetic management [1,2], including a careful review of electrolytes such as potassium, magnesium and calcium, which influence QT dynamics [3,4].

The patient was deemed at high risk for peripartum cardiac events due to her history of recurrent VT despite multiple interventions, her elevated emotional stress levels, highlighted by an ICD shock triggered by an anxiety episode early in pregnancy, and her markedly prolonged QTc on admission [1,2]. Caesarean delivery is preferred in high-risk LQTS patients [1].

A thorough preoperative evaluation included a medical history review, physical examination and medication screening to avoid QT-prolonging drugs. Beta-blockers were continued throughout the perioperative period as recommended [1-5]. A 12-lead ECG and laboratory tests, particularly serum electrolytes, were assessed and optimised, as electrolyte imbalances, such as hypokalaemia, hypomagnesaemia, and hypocalcaemia, can exacerbate QT prolongation [3,5]. Intravenous potassium and magnesium supplementation was initiated to correct persistent electrolyte abnormalities. Spironolactone was also added for its potassium-sparing effect to support the correction and stabilisation of serum potassium levels. Through these therapeutic adjustments, the patient’s QTc was successfully reduced to normal values before caesarean delivery, illustrating that QTc can be significantly improved with optimisation strategies.

Sympathetic modulation is a critical goal in preventing life-threatening arrhythmias in pregnant patients with LQTS [1-3]. However, there are no clinical trials defining optimal anaesthetic management in this population, and current evidence is largely derived from case reports and small series [1,3,4,7].

Several case reports have described the safe use of epidural or combined spinal-epidural anaesthesia in patients with LQTS [1]. In contrast, single-shot spinal anaesthesia is generally discouraged due to the potential for abrupt haemodynamic changes, which may increase the risk of malignant arrhythmias or other adverse cardiac events. Epidural anaesthesia is associated with more gradual haemodynamic changes and is therefore often preferred over spinal techniques [1,3]. Nevertheless, a major limitation of neuraxial anaesthesia is the risk of high sympathetic blockade, which may result in hypotension and bradycardia, parasympathetic predominance and the subsequent need for sympathomimetic agents that can further prolong the QTc interval [1,3,4].

Although epidural anaesthesia is typically safe [1,3,7], it was relatively contraindicated in this case due to severe anxiety and previous arrhythmic responses to emotional stress. In patients with LQT1, severe anxiety is a well-recognised trigger for arrhythmias [1,3,4], supporting the decision to pursue general anaesthesia. General anaesthesia was also chosen to allow better haemodynamic control and rapid intervention in the event of an acute cardiac episode. In addition, suspected foetal LQTS further increased maternal anxiety, reinforcing the choice of general anaesthesia as the preferred approach to mitigate stress-related arrhythmogenic triggers.

General anaesthesia for LQTS requires careful planning and execution. As referred to before, anxiety can precipitate arrhythmias; thus, anxiolytic premedication is recommended [3]. Midazolam was used, as it does not prolong QTc or disrupt cardiac conduction [3]. Normothermia, oxygen supplementation and normocapnia were ensured to avoid additional QT-prolonging triggers [3,8].

Sympathetic stimulation during induction and emergence was managed with opioids and lidocaine to blunt the sympathetic response [3,4]. Prophylactic intravenous magnesium sulphate (2-4 g) was also given to mitigate the risk of VT, as it can effectively prevent and terminate VT [3,5].

Pain is also a trigger to adrenergic response, so optimal analgesic management is required [3,4]. Opioids such as fentanyl or remifentanil blunt hemodynamic response and are considered useful and safe in patients with LQTS [4,8]. Remifentanil was chosen for its short half-life and rapid metabolism, with infusion discontinued just before umbilical cord clamping to minimise neonatal opioid exposure.

Propofol-based TIVA was utilised due to its favourable QT profile. Other intravenous anaesthetics, such as etomidate and thiopental, prolong the QTc interval more than propofol, and all halogenated volatile anaesthetics are also known to extend QTc [3,4,6].

Non-depolarising neuromuscular blockers are generally safe, while succinylcholine should be avoided due to its potential for QT prolongation and vagal-induced asystole [3,4,8]. Neuromuscular block reversal with sugammadex was preferred over anticholinesterase-anticholinergic reversal [3,8,9].

Postoperative nausea and vomiting prophylaxis should not be performed with first-generation 5-HT3 receptor antagonists or droperidol, as they can also prolong QTc [4,6]. Metoclopramide and dexamethasone can be safely administered in these patients [3,4]. Despite a high Apfel score, only dexamethasone was used to minimise QT-prolongation risks, with TIVA contributing as a nausea-reducing strategy.

Sulprostone and misoprostol were used as alternative uterotonics because oxytocin is considered potentially arrhythmogenic and should be avoided or used with caution in patients with LQTS, particularly when administered as a rapid intravenous bolus [4,5].

Given the risk of VT, continuous intra-arterial blood pressure and electrolyte monitoring were maintained throughout surgery [1,4].

For patients with an ICD, special precautions are required [1,3,4]. However, after cardiology consultation, it was determined that the ICD could remain active since the surgical site was infraumbilical and diathermy was not required. Defibrillation pads were positioned as a precaution, and a cardiologist was present throughout the procedure.

Postoperative management of patients with LQTS should include permanence in a postsurgical intensive care unit for at least 24h-48h [4]. The QT interval should be monitored, and the ICD should have the original programming setting restored [3,4]. Other goals of postoperative care include adequate pain control and a quiet environment [1,3]. Multimodal analgesia was the option, with regional techniques and analgesics from various pharmacological classes.

Close follow-up in the early postpartum period is crucial [1,2,5]. Therefore, monitoring by a cardiologist within the first weeks postpartum and then monthly for the first nine months is recommended to evaluate treatment efficacy, ECG changes and symptom progression [5].

## Conclusions

The uniqueness of this case lies not merely in the presence of LQT1, but in the convergence of multiple high-risk elements, including prior episodes of sustained VT, foetal conduction abnormalities and pronounced maternal anxiety, which collectively heightened perioperative risk. Regional anaesthesia was considered, but deemed inappropriate given the patient's severe anxiety and previous arrhythmic responses to stress. In the absence of established guidelines, this case underscores the importance of multidisciplinary collaboration and careful planning, with anaesthetic management tailored to the patient’s physiological and psychological profile to minimise arrhythmogenic risk.
